# The BRAIN Initiative data-sharing ecosystem: Characteristics, challenges, benefits, and opportunities

**DOI:** 10.7554/eLife.94000

**Published:** 2024-11-27

**Authors:** Sudhanvan Iyer, Kathryn Maxson Jones, Jill O Robinson, Nicole R Provenza, Dominique Duncan, Gabriel Lázaro-Muñoz, Amy L McGuire, Sameer A Sheth, Mary A Majumder

**Affiliations:** 1 Center for Medical Ethics and Health Policy, Baylor College of Medicine Houston United States; 2 Department of History, Purdue University West Lafayette United States; 3 Department of Neurosurgery, Baylor College of Medicine Houston United States; 4 Laboratory of Neuro Imaging, USC Stevens Neuroimaging and Informatics Institute, Keck School of Medicine of USC, University of Southern California Los Angeles United States; 5 Center for Bioethics, Harvard Medical School Boston United States; 6 Department of Psychiatry, Massachusetts General Hospital Boston United States; Monash University Australia; University of Oxford United Kingdom

**Keywords:** BRAIN initiative, data sharing, data archives, neuroethics, policy

## Abstract

In this paper, we provide an overview and analysis of the BRAIN Initiative data-sharing ecosystem. First, we compare and contrast the characteristics of the seven BRAIN Initiative data archives germane to data sharing and reuse, namely data submission and access procedures and aspects of interoperability. Second, we discuss challenges, benefits, and future opportunities, focusing on issues largely specific to sharing human data and drawing on *N* = 34 interviews with diverse stakeholders. The BRAIN Initiative-funded archive ecosystem faces interoperability and data stewardship challenges, such as achieving and maintaining interoperability of data and archives and harmonizing research participants’ informed consents for tiers of access for human data across multiple archives. Yet, a benefit of this distributed archive ecosystem is the ability of more specialized archives to adapt to the needs of particular research communities. Finally, the multiple archives offer ample raw material for network evolution in response to the needs of neuroscientists over time. Our first objective in this paper is to provide a guide to the BRAIN Initiative data-sharing ecosystem for readers interested in sharing and reusing neuroscience data. Second, our analysis supports the development of empirically informed policy and practice aimed at making neuroscience data more findable, accessible, interoperable, and reusable.

## Introduction

The BRAIN Initiative, led by the US National Institutes of Health (NIH), launched in 2013 (https://braininitiative.nih.gov/). The program involves 10 NIH institutes and numerous additional federal and non-federal affiliates. The BRAIN Initiative’s core strategy is to build and sustain research partnerships between academics and industry, geared toward an overall objective of ‘neurotechnology’ development for scientific discovery, medical diagnosis, and therapy (BRAIN 2025: A Scientific Vision, 2014).

Data sharing—sharing scientific data with other researchers before or as soon as possible after publication of related findings—has been an integral component of the BRAIN Initiative from its inception. Since 2017, the BRAIN Initiative has supported the development and maintenance of a distributed network of ‘data archives’, what we call a data-sharing ‘ecosystem’, which at present is composed of seven archives (Table 1). These differ in the data they host as well as in various other characteristics, such as upload and download procedures, data access levels and rules (tiers), and associated tools. The BRAIN Initiative’s 2019 Data Sharing Policy (NOT-MH-19-010) directs researchers receiving BRAIN Initiative funding to submit the data generated by their projects, once it has been de-identified to industry standards, to one of these archives every 6 months, at which point it will remain private until the first associated paper is published or the end of the award period, whichever comes first. This timeline is consistent with that in the current NIH-wide Data Management and Sharing (DMS) policy (NOT-OD-21-013), which came into effect in January of 2023 and presumably will drive further data flow into BRAIN Initiative-supported archives. The NIH, and some BRAIN Initiative archives, encourage investigators to seek as broad consent as possible for sharing data derived from humans, in accordance with IRB approval and barring any concerns regarding the possibility of data re-identification (Bannier et al., 2021; Markiewicz et al., 2021).

**Table 1.** BRAIN Initiative data archive ecosystem key statistics and related information. Sources: For Host Institution, Initial Funding Date, Total NIH Support, Administering ICO, and Funding ICO, information was taken from the NIH RePORTER entry for the most recent award for each archive, as of June 6, 2024 (https://reporter.nih.gov/) Total NIH Support for each archive was taken from the History section of RePORTER. A separate line of NIH funding, totaling $1,707,134, with NIMH listed as the awardee, has supported the development of OpenNeuroPET. Other information in the table was derived from the International Neuroscience Coordinating Facility (INCF) Infrastructure Portfolio (https://www.incf.org/infrastructure-portfolio) and archive websites. Where an archive landing page or its data portal did not offer a public ‘datasets‘ (or ‘dandisets’, in the case of DANDI) count, we used an available alternative such as the number of ‘projects‘ for which data are available. *Data current as of June 6, 2024. ICO = NIH Institutes, Centers, and Offices.

Archive	Host Institution	Initial Funding Date	Total NIH Support (USD)*	Administering ICO	Funding ICO	Data Types Hosted	Data Formats Supported	Publicly Available Datasets*
Brain Image Library (BIL)	Carnegie-Mellon University	2017	$6,767,625	NIMH	NIMH	Confocal microscopy	DICOM or NIfTI; for derived data, any format as long as primary data are in the required format	8418
Brain Observatory Storage Service and Database (BossDB)	Johns Hopkins University	2018	$5,133,219	NIMH	NIMH	Electron microscopy; x-ray microtomography	PNG; APNG; JPG; BMP; GIF; PSD	50
Data Archive for the BRAIN Initiative (DABI)	University of Southern California	2018	$6,160,745	NIMH	NINDS	Focus on invasive neurophysiology; houses all imaging and brain signal data	EDF, Biosemi (.bdf), BrainVision (.eeg,.vhdr,.vmrk), EEGLAB (.set,.fdt), Blackrock NeuroPort (.nev,.nsX), Intan (.rhd,.rhs), MATLAB files (.mat,.m); iEEG-BIDS, NWB, or DABI	110
Distributed Archives for Neurophysiology Data Integration (DANDI)	Massachusetts Institute of Technology	2019	$8,309,010	NIMH	NIMH	Cellular neurophysiology, neuroimaging, and microscopy	BIDS; NWB	640
NeuroElectroMagnetic data Archive and tools Resource (NEMAR)	University of California, San Diego	2019	$4,464,874	NIMH	NIMH	EEG, MEG	BIDS	297
Neuroscience Multi-Omic Data Archive (NeMO)	University of Maryland, Baltimore	2017	$9,347,683	NIMH	NIMH	Multi-omics	FASTQ; TSV; BAM; BIGWIG; MEX; LIST; BED; QBED; CSV; H5; BIGBED; LOOM; MTX; ASPX	49
OpenNeuro	Stanford University	2018	$7,254,848	NIMH	NIMH	MRI, PET, MEG, EEG, iEEG	BIDS	1076

In this paper, we provide an overview and analysis of the BRAIN Initiative data-sharing ecosystem, focusing on aspects germane to how data are shared and reused. We then discuss challenges, benefits, and future opportunities arising from this distributed data-sharing ecosystem, focusing on issues largely specific to sharing human data. Except where closely related to interoperability or functions that sharply distinguish the archives from one another, we do not discuss built-in analytical tools and instead direct readers to the cited literature.

For the technical details, we reviewed publicly available documents, websites, and articles (i.e., Delorme et al., 2022; Duncan et al., 2023; Hider et al., 2022; Markiewicz et al., 2021; Rübel et al., 2022; Subash et al., 2023). From June 2021 through June 2023, we also downloaded and uploaded test datasets, where possible. Finally, for challenges, benefits, and future opportunities, we conducted semi-structured interviews (*N* = 34) with diverse stakeholders including BRAIN Initiative and non-BRAIN Initiative-funded scientists, data stewards, law and ethics scholars and policymakers, patient advocates, and industry representatives (see Materials and methods). Our first objective is to provide a guide to the BRAIN Initiative data-sharing ecosystem for readers interested in sharing and reusing neuroscience, and especially human neuroscience, data. Second, we hope to support the development of empirically informed policy and practice options aimed at making neuroscience data more findable, accessible, interoperable, and reusable (FAIR) (Hendriks et al., 2022; Jwa and Poldrack, 2022; Poline et al., 2022; Sandström et al., 2022; Wilkinson et al., 2016).

## Results

### Overview

The seven archives of the BRAIN Initiative data-sharing ecosystem are currently the Brain Image Library (BIL), Brain Observatory Storage Service and Database (BossDB), Data Archive for the BRAIN Initiative (DABI), Distributed Archives for Neurophysiology Data Integration (DANDI), NeuroElectroMagnetic data Archive and tools Resource (NEMAR), Neuroscience Multi-Omic Data Archive (NeMO), and OpenNeuro. Table 1 summarizes some key statistics and related information, including each archive’s NIH funding to date, the data types and formats supported, and the current number of datasets hosted.

In the first seven sections below, we compare and contrast the BRAIN Initiative archives’ data submission and access processes, including citation rules and tiers of user access. In addition, we report on the archives’ connections, including where they do and do not work together and exchange information effectively (interoperability). In-principle data interoperability is achieved via standard data formats, for example, Neurodata Without Borders (NWB) (Rübel et al., 2022; Teeters et al., 2015) and the Brain Imaging Data Structure (BIDS) (Gorgolewski et al., 2016; Holdgraf et al., 2019; Milham et al., 2018; Niso et al., 2018; Pernet et al., 2019). The use of such standards facilitates pooling, re-analysis, and experimental replication. At the archive level, in-principle interoperability also includes the ability to find (i.e., by way of indexing and/or hyperlinking), access (i.e., download), and/or analyze data from one archive while working in another.

**Figure 1.**
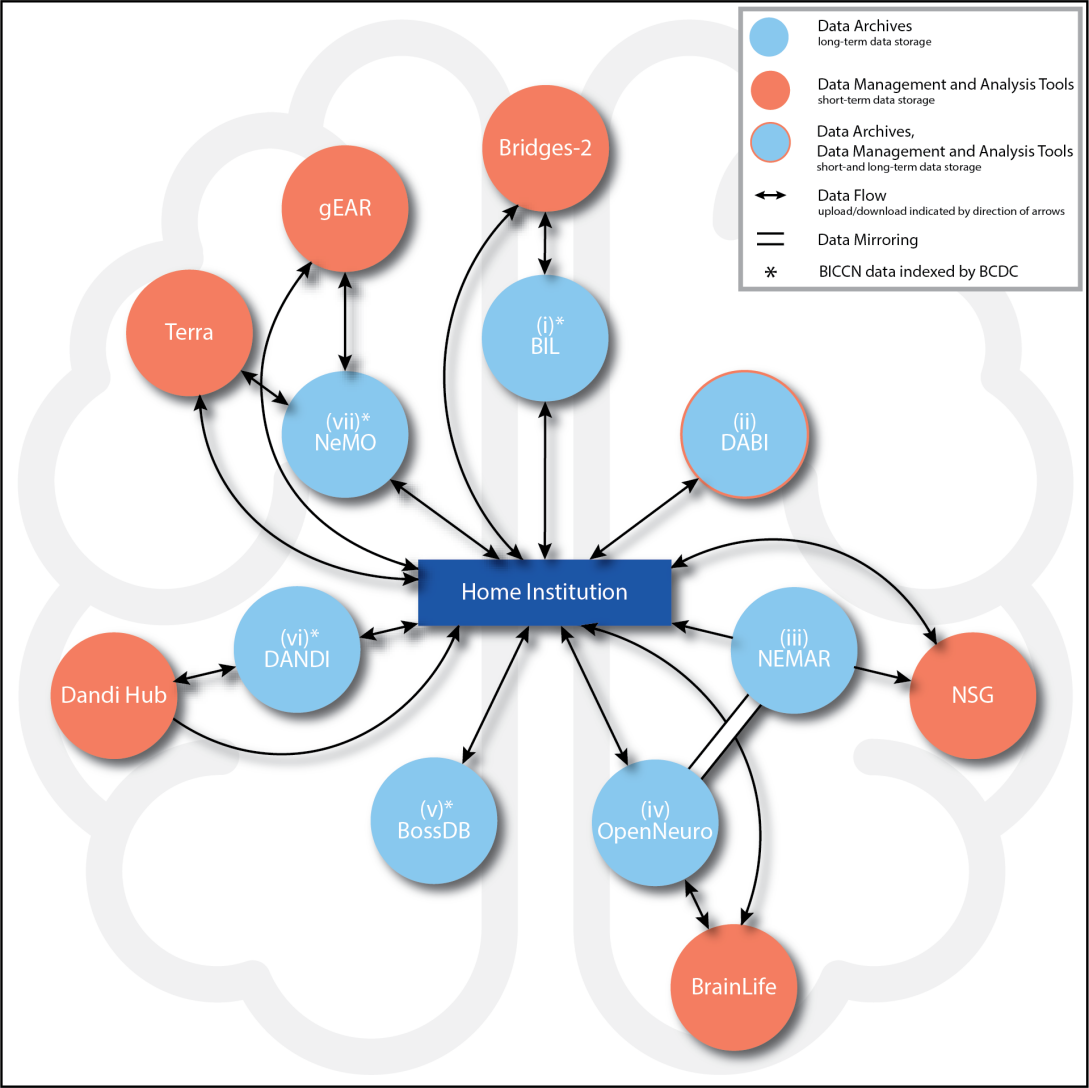
BRAIN Initiative data archive ecosystem connections. Summary of the main BRAIN Initiative data archive ecosystem connections currently enabling data upload, access (ability to download), analysis (which includes short-term data storage), and mirroring (identical copies of data across archives) among entities. Potential paths of data flow are represented by arrows, with the directions of flow (upload, download, or both) indicated by the directions of the arrows. All seven archives (light blue circles) are intended for long-term data storage. For the archives marked with an asterisk (*), data generated by the BRAIN Initiative Cell Census Network (BICCN) also are indexed by the Brain Cell Data Center (BCDC) at the Allen Institute in Seattle, WA. Clockwise from top: (**i**) Brain Image Library (BIL): From the data producer’s Home institution (hereafter, ‘Home’) (dark blue rectangle), data can be uploaded to or downloaded from BIL. BIL data can be uploaded to, analyzed, and downloaded from the Bridges-2 supercomputer (orange circle). (**ii**) Data Archive for the BRAIN Initiative (DABI): From Home, data can be uploaded to or downloaded from DABI. (**iii**) NeuroElectroMagnetic data Archive and tools Resource (NEMAR): From Home, data can be downloaded from NEMAR. From NEMAR or Home, data also may be uploaded to the Neuroscience Gateway (NSG), although analyzed data may only be downloaded back to Home (and not back to NEMAR). All NEMAR data are mirrored (double solid line with no arrows) in OpenNeuro, and data also can only be uploaded to NEMAR through OpenNeuro. (**iv**) OpenNeuro: Data can be uploaded to or downloaded from OpenNeuro from Home. From OpenNeuro or Home, users also may upload and download data to BrainLife, a cloud-computing analysis environment. Through the data mirror, all OpenNeuro datasets are accessible on NEMAR. (**v**) Brain Observatory Storage Service and Database (BossDB): From Home, data can be uploaded to or downloaded from BossDB. (**vi**) Distributed Archives for Neurophysiology Data Integration (DANDI): From Home, data can be uploaded to and downloaded from DANDI. From Home, data analyzed in DANDI Hub also may be downloaded directly, but uploads to this analysis environment must always take place through DANDI. (**vii**) Neuroscience Multi-Omic Data Archive (NeMO): This archive is the primary repository for genomic, transcriptomic, and epigenetic data generated by the BICCN. From Home, data can be uploaded to or downloaded from NeMO. NeMO also streamlines user access to Terra and gEAR. By way of the BCDC, users also may link directly to Terra workspaces associated with NeMO projects. All descriptions current as of June 5, 2024.

To emphasize the current paths of interoperability, Figure 1 summarizes the main connections enabling data submission and access procedures and mirroring (identical copies of data across archives) in the BRAIN Initiative data-sharing ecosystem. The figure also indicates prominent connections to external data management, indexing, and analysis tools, including the BRAIN Initiative Cell Census Network (BICCN) (https://biccn.org/). While not an official BRAIN Initiative data archive, the BICCN’s Brain Cell Data Center (BCDC), run by the Allen Institute for Brain Science in Seattle, WA, provides “public access to and organization of the complex data, tools, and knowledge derived by the BICCN.” Data appearing in four of the seven BRAIN Initiative archives (NeMO, BossDB, BIL, and DANDI) are indexed and findable on the ‘Brain Knowledge Platform’ provided by BCDC. In the following sections, we provide details on each of the BRAIN Initiative archives in terms of submission, access, interoperability, and associated tools.

### BRAIN Initiative data archives

#### Brain Image Library

The BIL (brainimagelibrary.org) is a data archive for confocal microscopy. It operates as a partnership between the Biomedical Applications Group at the Pittsburgh Supercomputing Center (PSC), the Center for Biological Imaging at the University of Pittsburgh, and the Molecular Biosensor and Imaging Center at Carnegie Mellon University in Pittsburgh, PA. BIL incorporates Bridges-2 (https://www.psc.edu/resources/bridges-2/), a supercomputer, for conducting analyses. Data uploaded to the BIL is managed under a tiered-access system, with the ability for data uploaders to designate secondary data access as public, restricted, embargoed, or ‘pre-release’, with the latter three options falling under ‘controlled access’ (https://www.brainimagelibrary.org/restrictedaccess.html; for tiered-access models more generally, see Broes et al., 2018). ‘Pre-release’ datasets have not yet gone through the full BIL data submission process. Data uploaded to BIL must include descriptive metadata. BIL encourages data users to contact producers for potential collaborations, and it lists multiple ways in which BIL and data producers can be cited in publications. BIL’s connection to the Bridges-2 supercomputer sets it apart from other BRAIN Initiative archives. BIL data generated by the BICCN also are indexed and findable through the BCDC.

#### Brain Observatory Storage Service and Database

Established and managed by Johns Hopkins University in Baltimore, MD, the BossDB (bossdb.org) is an archive for 3D and 4D electron microscopy (EM) and x-ray microtomography data (Hider et al., 2022). Data uploaded to BossDB is managed under a tiered-access system, with classification of datasets as either public or controlled. As of this writing, there are no specific requirements for citing data hosted on BossDB.

In 2016, the US National Science Foundation (NSF) funded a series of strategy meetings to discuss how to use emerging cloud resources for analyses of large-scale brain data, such as that generated by EM and x-ray microtomography (Vogelstein et al., 2016; Wiener et al., 2016). BossDB emerged in part from these discussions. In addition, BossDB receives support from the Intelligence Advanced Research Projects Agency’s (IARPA) Machine Intelligence from Cortical Networks (MICrONS) program, a federal research initiative investigating how brain biology can inform artificial intelligence (https://www.iarpa.gov/research-programs/microns). The Amazon Web Services (AWS) Public Datasets project, which covers the cloud storage costs for public datasets (https://registry.opendata.aws/), also supports BossDB.

Today, BossDB’s ability to support storage and analyses of especially large datasets—up to multiple petabytes at once—continues to set it apart from the other BRAIN Initiative archives. Additionally, because BossDB was designed for the cloud, the archive can manage cost and performance without sacrificing storage and analysis; as is true more generally for cloud-based archives, cloud operation ensures that the archive pays only for storage that is utilized, as opposed to paying for space that may not be fully used (Vogelstein et al., 2016). BossDB data generated by members of the BICCN are indexed and findable through the BCDC.

#### Data Archive for the BRAIN Initiative

The DABI, residing at the Laboratory of Neuro Imaging (LONI) at the University of Southern California (USC), holds invasive human electrophysiological (i.e., iEEG) and associated clinical, imaging, pathology, behavioral, and demographic metadata (https://dabi.loni.usc.edu/about/overview; Duncan et al., 2023). Currently, DABI offers a collaborative annotation tool that allows users to annotate their brain signal (multi-neuron electrical) data in real time (https://dabi.loni.usc.edu/analysis/example-sync), with future plans to implement signal and imaging analysis tools.

DABI focuses on invasive neuro-electrophysiology but houses all imaging and signal data. The archive works with providers to offer extensive personalized support at the time of upload, tailored to the data type. Backup mechanisms preserving raw and processed data ensure data integrity throughout. DABI also uses a tiered-access system where datasets can be classified as public or private. All data requests require a DABI account, and some public and private datasets require a data use agreement. Private datasets can only be accessed following a formal request and approval from a dataset’s owner. According to DABI’s Publication Policy (https://dabi.loni.usc.edu/about/publications), data users must agree not to reference any of the preliminary analyses provided in DABI in their publications. Moreover, this policy states that:

“If DABI users are granted access to data and wish to publish results of analysis performed on the data, they must cite the Data Provider (per the agreed upon conditions) and include the following in their acknowledgements: ‘Data used to perform this analysis was accessed from the Data Archive for the BRAIN Initiative with support from the National Institutes of Health under Award Number R24MH114796.’”

DABI also tracks publications that make use of its data, which range from more granular research reports to studies of how data infrastructures, such as the existence of archives, influence the development of therapies (Chen et al., 2021; Magnotti et al., 2020; Mujica-Parodi et al., 2020; Sendi et al., 2021).

The front-end user support that DABI offers is one of the archive’s distinctive features. Another is the choice that it affords data generators between centralized and federated storage. At present, no streams of interoperability, such as reciprocal data access, exist between DABI and the other BRAIN Initiative archives.

#### Distributed Archives for Neurophysiology Data Integration

The DANDI is a multi-institutional effort, supported by the BRAIN Initiative and the AWS public dataset programs, to house versioned data and collaborate across research sites (https://www.dandiarchive.org/). This archive was built around NWB and a commitment to the FAIR Principles. It stores electrophysiology, optophysiology, behavioral time series, and immunostaining data. Cellular neurophysiology data must be in NWB, and neuroimaging data must be BIDS compliant (or use the BIDS extension for microscopy for immunostaining) (https://www.dandiarchive.org/handbook/30_data_standards/).

DANDI is managed under a tiered-access system, where datasets are classified up front as either fully public or embargoed. The embargo option allows data producers to easily adhere to the BRAIN Initiative’s Data Sharing Policy, which permits an embargo of up to 6 months before data are released for public access. Additionally, data producers have the option of controlling who has access while the data are still embargoed and when the data are released. Publicly available code can be used to access public data and upload to DANDI, while requests for access to embargoed data must go through the DANDI web interface. When citing data, users should cite the unique DANDI identifier associated with the dataset, assigned at the time of successful data upload.

Data files uploaded to DANDI and analyzed in DANDI Hub, the DANDI-associated analysis workspace, must be formatted as a ‘dandiset’, “a collection of assets (files and their metadata) and metadata about the collection” (https://www.dandiarchive.org/handbook/10_using_dandi/). Users may work with any NWB- or BIDS-compliant data in DANDI Hub. Data from multiple archives in NWB or BIDS formats can be uploaded as dandisets. However, there is currently no way to access data from other archives within DANDI itself. DANDI data generated by the members of the BICCN are indexed and findable through the BCDC.

#### NeuroElectroMagnetic data Archive and tools Resource

The NEMAR operates as a partnership between OpenNeuro’s Stanford-based (Palo Alto, CA) team and the University of California, San Diego (UCSD) (https://nemar.org/). It is an archive for magnetoencephalography (MEG), electroencephalography (EEG), and intracranial electroencephalography (iEEG) (collectively, neuroelectromagnetic, or NEM) data, supporting “the creation, maintenance, analysis, and cross-study mining” of such data and the “seeding and growing [of] ‘minable’ archives of NEM data in the OpenNeuro resource” (Delorme et al., 2022). The great expense of collecting NEM data strengthens the argument for maximizing access to it (Feinsinger et al., 2022; Mercier et al., 2022). NEMAR facilitates analyses of its data with supercomputing resources available through a partnership with the NSF-funded Neuroscience Gateway (NSG) (https://nemar.org/nsg_for_nemar), which provides users free supercomputing time as well as streamlined access (through an integrated portal) to publicly available and some commercial software tools (https://www.nsgportal.org/; Martínez-Cancino et al., 2021; Sivagnanam et al., 2020).

NEMAR is unique among the BRAIN Initiative archives in its approach to data upload (https://nemar.org/openneuro_and_nemar_relationship). Datasets cannot be uploaded directly, but rather only through OpenNeuro, at which point data appears in NEMAR within 24 hr. Identical copies of data (data mirrors) thus exist across the archives. Data may be designated as public or private, and private data are accessible only to designated users. OpenNeuro checks all datasets for BIDS compliance, enabling data interoperability between archives employing BIDS. NWB datasets will pass OpenNeuro’s validation, and NWB-BIDS interoperability is improving (https://neurodatawithoutborders.github.io/nwb_hackathons/HCK10_2021_Remote/projects/BIDS/). Requirements for citing datasets in NEMAR mirror those in OpenNeuro (see below).

To date, NEMAR is the only BRAIN Initiative archive to have been founded with the express purpose of interoperability with another BRAIN Initiative archive. NEMAR was always imagined as a “gateway to OpenNeuro for human electrophysiology” (Delorme et al., 2022), filling an important gap by providing a centralized archive of NEM data allowing direct comparison of NEM brain dynamics and modalities (e.g., MEG and EEG) (Delorme et al., 2022). NEMAR remains the largest archive of its kind. Other, smaller NEM archives exist, though they do not share a data standard.

#### Neuroscience Multi-Omic Data Archive

Based at the Institute for Genome Sciences at the University of Maryland School of Medicine in Baltimore, the NeMO is the primary repository for genomic, transcriptomic, and epigenetic data, for humans and model organisms, generated by the BICCN (https://nemoarchive.org/). NeMO’s web-based platform offers access to Terra (https://terra.bio/), an open-source, cloud-based repository of tools (O’Connor et al., 2017; Yuen et al., 2021). Through NeMO Analytics (https://nemoanalytics.org/), NeMO users also may analyze datasets in the archive in the gEAR portal (https://umgear.org/), “a single platform for data deposition, display, analysis, and interrogation” (Orvis et al., 2021) that can link out to species-specific gene annotations available on a diversity of online platforms.

Data submitted to NeMO is hosted at the NeMO website and can be categorized as public, embargoed, or restricted. The archive defines restricted data as: “to be distributed only to an approved group of users due to consent restrictions, e.g. human data” (https://nemoarchive.org/resources/data-download). At present, there are no requirements for citing NeMO datasets, although each NeMO dataset has a unique identifier that can be cited, and some public datasets already are associated with publications (https://data.nemoarchive.org/publication_release/).

To enable the integration with Terra, NeMO relies on support from the Broad Institute in Cambridge, MA and the Google Cloud Platform (GCP), the latter of which keeps a copy of all NeMO data to facilitate the creation of NeMO projects in Terra.

#### OpenNeuro

Managed by the Stanford Center for Reproducible Neuroscience, OpenNeuro is a data archive that stores MRI and other neuroimaging data using BIDS (openneuro.org, Markiewicz et al., 2021). Developed initially out of the OpenfMRI archive, OpenNeuro is part of a long line of efforts—which reach back at least to the establishment of the fMRI Data Center in 1999—to support data sharing in neuroimaging communities (Poldrack et al., 2013; Van Horn and Gazzaniga, 2002). Like DANDI and NeMO, OpenNeuro is explicitly committed to the FAIR Principles, and all its public data are released under a CC0 license that places no restrictions on how the data can be reused (https://creativecommons.org/share-your-work/public-domain/cc0/). In turn, as so many of its datasets are openly available, OpenNeuro participates in the AWS Public Datasets project (https://registry.opendata.aws/openneuro/).

Data uploaded to OpenNeuro are managed under a tiered-access system, with the ability to classify data as either public or controlled. OpenNeuro account holders may analyze data via OpenNeuro’s partnership with the cloud-based environment, Brainlife.io (Hayashi et al., 2024). As noted above, data intended for NEMAR also must be funneled through OpenNeuro. Moreover, while OpenNeuro’s standard policy is to make data publicly available “after a grace period of 36 months counted from the first successful version of the dataset” (https://openneuro.org/faq), allowing for analyses and associated publications, authors can apply for up to 12 additional months (and thus a 4-year total embargo).

OpenNeuro only accepts BIDS-compliant data free, in the case of human data, of the 18 HIPAA identifiers (https://www.hhs.gov/hipaa/for-professionals/special-topics/research/index.html). All data uploaded must pass OpenNeuro’s BIDS validator. As some MRI data contain structural information that could be a basis for identifying an individual, authors must use suitable programs to remove any defining facial features before upload. Finally, data generators must obtain the ethics permissions and informed consent necessary for sharing human data under their chosen tier of access, with OpenNeuro encouraging all data generators to use the language available in the Open Brain Consent (Bannier et al., 2021). Examples of how to cite datasets are provided, and users are encouraged to reference the dataset numbers provided by the archive in any follow-on publications (https://openneuro.org/cite).

OpenNeuro stands out from the other BRAIN Initiative archives in its deliberate interoperability with NEMAR. Additionally, the size and multi-dimensionality of many of its datasets set it apart, as does the degree to which the archive has tracked the reuse of its own data. Many datasets in OpenNeuro fall into the category of ‘big’ data along three axes: ‘width’ (the number of data subjects), ‘breadth’ (the number of phenotypes measured), and ‘depth’ (the number of measurements per individual) (Markiewicz et al., 2021, p. 7). OpenNeuro’s leaders have reported actual reuse of the archive’s data in 165 publications through 2021, resulting in savings of thousands of hours of patient visits and research costs in the millions (Markiewicz et al., 2021).

### Challenges, benefits, and future opportunities

Our first purpose in this paper has been to provide a guide for readers interested in sharing and using BRAIN Initiative-generated and other neuroscience data. In Figure 1, we summarize paths of data upload, download, mirroring, and relationships with tools germane to sharing and reusing data within the seven BRAIN Initiative-funded data archives. For this discussion, we have drawn on published reports (i.e., Delorme et al., 2022; Duncan et al., 2023; Hider et al., 2022; Markiewicz et al., 2021; Rübel et al., 2022; Subash et al., 2023), the archives’ websites, and our own experiences (SI) downloading and uploading test datasets where possible. Relatedly, our second purpose is to identify and discuss policy-relevant challenges, benefits, and future opportunities emerging from the distributed BRAIN Initiative archive ecosystem, adding to ongoing efforts promoting empirically informed policy and practices aimed at making neuroscience data more FAIR (Hendriks et al., 2022; Jwa and Poldrack, 2022; Poline et al., 2022; Sandström et al., 2022; Wilkinson et al., 2016). Below, we highlight some of these aspects, incorporating data from semi-structured interviews with diverse stakeholders (*N* = 34) and focusing on challenges largely specific to sharing human data. These challenges relate to archive interoperability and data stewardship.

#### Interoperability challenges

Several challenges related to data and archive interoperability are apparent in the BRAIN Initiative’s distributed archive ecosystem. Perhaps the most pressing is that training in adhering to existing data standards, such as NWB and BIDS, and processes for data submission in these formats are not built into most laboratory workflows, a challenge that only multiplies when research groups want (or are required) to submit data to multiple archives that employ different data standards and/or upload procedures. As one data steward we interviewed noted: “[F]rankly, there’s a lot of idiosyncrasies to different archiving systems.” (Interview 303). This individual also suggested that “[I]nfrastructurally… [in your lab] you need someone who can help others [share data in one of the BRAIN archives], instead of making everyone learn how to do it for every different type of archive system.” (Interview 303). Yet, whether for all laboratory members or just designated personnel, the time and resources required to acquire the expertise needed for sharing, and to overhaul existing data generation and annotation procedures, must be built into the data-sharing costs for any research group (Bush et al., 2022). This point was corroborated in a 2022 survey of 1190 BRAIN Principal Investigators, which identified the limited standardization and norms for data acquisition, formatting, and description,” “heterogeneity of data types,” and the costs and time involved in data sharing” as the three predominant barriers to data sharing within the BRAIN Initiative (Hendriks et al., 2022). Again, it is worth emphasizing that these barriers are higher with submission to multiple archives.

Moreover, some uncertainties associated with the enforcement of data-sharing policies, which promote compliance with data standards, add a layer of complexity. For instance, the BRAIN Initiative Data Sharing Policy requires that researchers funded through the BRAIN Initiative use the BRAIN informatics infrastructure, including the BRAIN Initiative-funded archives and associated standards. Nevertheless, the policy does not describe sanctions for non-compliance. The 2023 NIH DMS policy and related guidance establish that compliance will be reviewed during regular reporting intervals for a grant, along with annual Research Performance Progress Reports (RPPRs). Furthermore, the 2023 NIH DMS Policy states that non-compliance with an approved DMS plan may result in enforcement actions, including addition of special terms and conditions to or termination of the award, and may impact future funding decisions for the recipient institution. It is unknown, however, how fully NIH officials will embrace these enforcement powers. The effects that any such actions would have on adherence to data standards also are unclear.

The experiences within the US National Institute of Mental Health (NIMH) in the evaluation of DMS plans might offer some insights into how data-sharing policies could affect compliance with data standards in the BRAIN Initiative moving forward. For example, the NIMH has centralized the evaluation of all DMS plans, although the assigned program officer still makes the final determination of acceptability (as laid out in the NIH’s DMS policy). Greg Farber, Director of the NIMH’s Division of Data Science and Technology, noted in a Town Hall focused on DMS in December 2023 that in the NIMH’s October 2023 Council Round, 14% of the 157 DMS plans submitted to the NIMH involving human subjects got a C and 22% got a D (https://sharing.nih.gov/data-management-and-sharing-policy/resources/learning?policy=DMS). Plans assigned a score of ‘C’ or ‘D’ will typically have to be revised or completely redone, respectively. Yet, these outcomes were better than for research with non-human subjects, and, as also noted in the above Town Hall, NIMH staff believe this contrast is tied to the influence of archives:

“If you’re going to deposit data to a well-established archive that has longstanding clear rules about how to deposit data and timelines and things like this, that...DMS plan is pretty easy to write because you just have to say, ‘Well, I’m going to do what the archive requires, I’m going to use these data dictionaries, you know, I’m going to do this, that, and the other thing.’”

Thus, while a distributed archive ecosystem has created some interoperability challenges, including for groups looking to submit data to multiple archives, the archives themselves have proven integral in enforcing the use of relevant data standards at the time of data sharing.

Additionally, the NIMH Data Sharing Policy (NOT-MH-23-100) requires that researchers funded by the NIMH use the NIMH’s own informatics infrastructure, which includes submitting all data to the NIMH Data Archive (NDA) unless NIMH stipulates otherwise during negotiation of the award terms and conditions. The fact that a substantial fraction of BRAIN Initiative awards are administered by the NIMH has created friction for data generators, some of whom expressed concern about compounded sharing efforts and the chances of errors being introduced in different copies of the same data. As one investigator noted: “[The NIMH] won’t accept sharing the data through DABI. …[T]hey have a whole other pipeline at NIMH….” (Interview 305). A closely related policy issue, although not technically one of interoperability, is the potential confusion that multiple archives can cause for investigators in terms of the ‘right’ or ‘best’ places to upload data. One policymaker noted: “It’s not clear which single database [BRAIN Initiative investigators] are then beholden to.” (Interviewee 318). “[Y]ou have somebody who’s got data that falls into three or four of these buckets, which one [archive] are they imposing [the policies upon], all of them?” (Interviewee 318).

To summarize, likely the most pressing interoperability challenge associated with the BRAIN Initiative’s distributed archive ecosystem relates to standards, including the labor of adhering to and enforcing them and the layers of complexity that individual agency standards add to the data-sharing process.

Additionally, those paths of in-principle interoperability that do exist in the BRAIN Initiative data-sharing ecosystem, whether among data or archives, could fail in practice, and in ways that may not even be predictable until the archives and their connections are more heavily trafficked. “That’s the next big question. Everyone’s trying to put data in. But how many people are using it?” one policymaker noted (Interview 125). Consequently, constant checking, use, and maintenance are required to ensure smooth operation: “[T]he biggest issue is that things break all the time and web infrastructure changes all the time” a data steward noted (Interview 320). The problem is in part traceable to the history of how the data-sharing ecosystem developed and evolved, given that the BRAIN Initiative archives arose for the most part in response to the needs of individual research communities (i.e., neuroimaging). One policymaker recalled that, as early as the 1990s, some neuroscientists already were discussing ways in which the Internet, then new, could be employed to share data: “Do we have one gigantic database? And does that database cover brain connectivity? Or do we, you know, build a bunch of databases for different data types?” (Interview 116). The distributed network ultimately adopted within the BRAIN Initiative meant that interoperability between the archives became a challenge to solve after the launch of each archive, rather than one built into the system up front. “[T]he time was right under the BRAIN Initiative to build archives,” one data steward noted (Interview 319). Yet, at first, “…there were no identifiers...there was no metadata. …[W]e had to really work on bringing those things together” (Interview 116).

To complicate matters, since 2017, the BRAIN Initiative archives, and their paths of interoperability, have evolved alongside processes of data standards development and implementation. Thus, data curation (i.e., is all data claiming to be NWB- or BIDS-compliant actually so?) persistently has been tangled with the work of building lines of communication between archives (i.e., are data that are supposedly accessible from an archive actually accessible?). In addition, if more archives were to be added to the ecosystem, more standards could emerge. The more data, archives, and standards that exist, the more maintenance that is required for reliable, functional interactions. In turn, as noted, there is always the concern that paths of in-principle interoperability can fail in practice.

Finally, we have identified at least one node in the distributed BRAIN Initiative data-sharing ecosystem that, were it to fail, could lead to significant disruptions of research. Aside from the paths of interoperability that have been built deliberately into OpenNeuro and NEMAR, the BCDC indexes, and thus makes findable by hyperlink, the data generated by the BICCN that also is available within four of the BRAIN Initiative archives (NeMO, DANDI, BIL, and BossDB) (Figure 1). Should this functionality be interrupted, secondary users could have trouble finding the large volume of data generated by the BICCN member institutions.

At least some of these interoperability issues are likely to be solved in the not-too-distant future. For example, while not entirely stemming from the existence of multiple BRAIN Initiative archives (as even a central database can support multiple standards), the challenge of making separate data standards themselves interoperable is receiving considerable attention from data stewards and neuroscientists (Markiewicz et al., 2021; Poldrack et al., 2024; Rübel et al., 2022). In addition, the efforts of data stewards and standards developers in launching and improving data standards, providing training for users, and beginning to forge paths of interoperability so far have been facilitated by a number of data resource selection, evaluation, and self-study tools keyed not just to the FAIR Principles, but also to similar criteria, such as the TRUST (Transparency, Responsibility, User focus, Sustainability, and Technology) Principles (Donaldson and Koepke, 2022; FAIR Data Maturity Model Working Group, 2020; Lin et al., 2020; Murphy et al., 2021; Poline et al., 2022; Sandström et al., 2022; Wilkinson et al., 2016).

As another illustration, participants in the NIH Cloud-Based Platform Interoperability Community/Governance Working Group recently published the Secure and Authorized FAIR Environment (SAFE) framework specifically to facilitate interoperability between cloud platforms (Grossman et al., 2024). Features include a system of authorized platform identifiers and authorized platform networks with their own identifiers; an API that exposes metadata relevant to the scope of appropriate sharing; and a platform governance process along the lines of the database of Genotypes and Phenotypes (dbGaP) (https://www.ncbi.nlm.nih.gov/gap/) or the National Institute of Standards and Technology (NIST) 800-53 Security and Privacy Controls for Information Systems and Organizations framework at the Moderate Level (Dempsey et al., 2014).

The International Neuroinformatics Coordinating Facility (INCF) also has emerged as a leader in facilitating interoperability among standards and archives and providing training, resources, and validation of standards in terms of (for example) metadata adaptability and the potential for widespread adoption (https://www.incf.org/). As a final example, the International Brain Initiative (IBI), a collective dedicated to international governance for neuroscience data, is developing resources to help investigators and other stakeholders navigate the myriad ethical, legal, regulatory, and policy frameworks affecting the work of large neuroscience projects (Eke et al., 2022). These include the US BRAIN Initiative but also other large-scale projects around the globe, such as the EU Human Brain Project (HBP) (Amunts et al., 2019) and similar efforts in Canada, China, Japan, Korea, and Australia.

These resources will continue to assist data stewards and generators. Yet, scientists and policymakers also will need to continue to foster the benefits of having multiple, linked archives, to balance out the interoperability-related challenges this network architecture imposes.

#### Data stewardship challenges

In addition to interoperability challenges, the distributed network of BRAIN Initiative archives raises certain ‘stewardship’ issues (Lin et al., 2020; Wilkinson et al., 2016). In ethics, ‘stewardship’ refers to the responsible management and supervision of resources of value to communities. Thus, ‘data stewardship’ refers to the responsible management of valuable data. Alongside the challenges of generating, using, and sharing FAIR data detailed above, two further stewardship challenges have emerged within the BRAIN Initiative data-sharing ecosystem: securing stable, long-term funding (i.e., sustainability) and grappling with the limits of informed consent for sharing of human data.

Since 2017, the NIH has made a significant investment in the BRAIN Initiative archives (Table 1). Funding for some has decreased over time, while others have seen their funding fluctuate, remain relatively stable, or increase. As is clear above, while some costs are specific to the planning and launch of an archive, continuing costs include more than just payment for storage. They also incorporate the labor required for responding to security threats; data curation; the development and maintenance of tools and resources (such as the NSG); and customer service. “It’s like rebuilding a raft as you’re floating on it,” one data steward noted, referring specifically to the evolving, expensive, and multi-faceted process of archive maintenance (Interview 320). Archive personnel must at a minimum adapt to this changing environment. Most data stewards, however, aspire to improve their user interfaces and available tools over time.

In turn, while the BRAIN Initiative’s commitment to infrastructure has quelled some fears, data stewards especially still worry about the prospects of obtaining long-term, sustainable funding for their archives. The NIH’s 2018 Strategic Plan for Data Science noted problems created by supporting “data *resources* using funding approaches designed for research *projects*” (https://datascience.nih.gov/nih-strategic-plan-data-science). The BRAIN R24 Funding Opportunity Announcements provided support for the existing archives (https://grants.nih.gov/grants/guide/rfa-files/RFA-MH-17-255.html; https://grants.nih.gov/grants/guide/rfa-files/RFA-MH-19-145.html). Yet, there remains the question of sustainability once the BRAIN Initiative transitions to a new phase: The original conception was focused on a 10-year time frame ending in 2025 (BRAIN 2025: A Scientific Vision, 2014), and special funding was allocated to the Initiative in 2016 under the 21st Century Cures Act through 2026 (https://www.nih.gov/research-training/medical-research-initiatives/cures). In 2024, BRAIN Initiative funding through the 21st Century Cures Act was significantly reduced, creating resource challenges for existing and new initiatives.

Stable funding helps archives with recruitment and retention of qualified staff. Relatedly, it helps incentivize data sharing, as submitters know that their investment in preparing data for upload will not be wasted by that archive ceasing to exist. While the latter point could apply both to distributed and centralized systems, avoiding disruption of a distributed ecosystem requires continued support for multiple archives. A business model that relies on user fees paid by data depositors and/or secondary users is conceivable, yet it inherently disadvantages potential users from lower-resourced institutions and countries. In the words of a 2017 OECD report, “willingness to pay is constrained by capacity to pay” (OECD Global Science Forum, 2017). Also, even those with the capacity to pay may be disinclined to do so if they can find similar data for free, as evidenced by the failure of efforts like BRCA Share (Bollinger et al., 2019). Thus, it is likely that the leaders of the BRAIN Initiative archives will continue to look to the NIH for funding to support their existence, although data stewards, such as those leading OpenNeuro and DABI, do engage in continuity planning (Duncan et al., 2023; Markiewicz et al., 2021). Ideally, such contingency plans will obviate the need to consider charging for access to data if sustainable NIH funding dries up. However, they are unlikely to maintain current levels of service and innovation.

Second, there is the stewardship challenge of the limits of informed consent for sharing human data in a distributed archive ecosystem. The ethical issues embedded in informed consent for neuroscience research is an active area of study in neuroethics (i.e., Cabrera, 2021; Feinsinger et al., 2022). Existing studies in relation to data sharing emphasize the impossibility of ‘future-proofing’ consent due to uncertainties in articulating the risks that could be involved (Bannier et al., 2021; Hendriks et al., 2022; Lee and Majumder, 2022). In part because one of the goals of sharing in a public archive is to facilitate data pooling, for example, it is likely impossible to predict all the ways in which shared data might be used, not to mention the inferences that could be made because of those uses. “[I]f you have different types of data that you’re combining, and then layering on top of that new approaches for analyzing those data with AI... I’m not confident that our informed consent can really capture what might be possible,” one policymaker noted (Interview 318). Moreover, the more interoperable and annotated neuroscience data become—a scientifically desirable outcome—the higher the risk for misuse, such as through re-identification: “[W]e want to get to a place where the data is incredibly interoperable and can be aggregated…” one legal and ethics scholar noted (Interview 324), yet, “…when we do that, we have a prime target for misuse.”

These more general challenges are compounded given the multiple BRAIN Initiative-supported archives. This is because consent provided by participants for certain tiers of secondary access to their data in one archive may not be enforceable across all the archives in the network. As interoperability between data and archives improves, this challenge will only grow more acute. “I don’t think we really talk about linking data and what it actually means,” one policymaker noted (Interview 316); “…if you wanted to link databases, you need to get informed consent for it. Because it actually makes people aware of what you're actually doing with the data.”

Harmonizing informed consents for the various tiers and rules of access across the BRAIN Initiative also likely will present a persistent and evolving challenge, both logistically and ethically. Currently, BRAIN Initiative-funded investigators are encouraged by the NIH (NOT-MH-19-010), and by some BRAIN archives, to seek as broad consent as possible for sharing their de-identified human data in public archives, if in accordance with IRB approval, and barring any concerns regarding the possibility of re-identification (Bannier et al., 2021; Markiewicz et al., 2021). A representative of the National Institute for Child Health and Development (NICHD), another BRAIN Initiative institution, noted in the December 2023 DMS Town Hall that a specific justification would be expected for any limits on the sharing of de-identified data as stipulated in DMS plans submitted to the Institute. For example, if an investigator were to reference a small sample size in such a justification, NICHD would expect an explanation of the IRB’s role in that determination.

Especially as the NIH’s new DMS policy promotes increased sharing across all fields, and as is the case in other fields (i.e., Garrison et al., 2016; Office of Science Policy, 2015), this area will remain a topic of policy concern.

#### Benefits and future opportunities

Despite these interoperability and stewardship challenges, there are benefits to the BRAIN Initiative’s distributed data-sharing ecosystem, and the model offers distinct opportunities for improvement as time goes on. Perhaps the most obvious benefit, and the one most frequently noted in our interviews, is the ability of individual archives to adapt smoothly to the needs of specific research communities. “I like the... model that the BRAIN Initiative has taken, in part because the archives that they are funding are all investigator-driven. They’re being developed by people in the field” one data steward noted (Interview 320). As mentioned above, a distributed infrastructure can avoid some of the technical and logistical challenges often associated with central servers, such as storage limitations and coarseness in responding to data-specific upload, download, and analysis issues (Rootes-Murdy et al., 2022; The Global Alliance for Genomics and Health, 2016; White et al., 2022). Moreover, responses to changes in the overall landscape—due to the possible addition of archives and standards, emerging security threats, experimental or technological innovations leading to unprecedented volumes or types of data, or changes in user preferences, for example—emanate from the bottom up rather than the top down. In other words, distributed archives can respond nimbly to user needs, presumably better than some more centralized systems, largely because the arrows of responsiveness flow from field experts directly to archives tailored to and run by them.

Relatedly, there are benefits to enforcing data standardization in a distributed network. Some aspects of data sharing, such as post-submission curation, may in principle be easier to enforce uniformly in a centralized archive. “[I]f you look at a lot of the standards, where they get implemented are at the repository levels, right?” a policymaker we interviewed noted (Interview 116). “[I]t’s the repositories that say: ‘Well, you need to have this metadata.’ So, once you have a mandate, like BRAIN does, that says, ‘you will put them into this repository,’ and the repository says, ‘and you’re not going to be able to put them in there until such a time as you have this information,’ you start to have something that can gain some traction” (Interview 116). On the other hand, archives emerging from specific research communities, such as OpenNeuro, are built with tailored purposes and users in mind, and are thus perhaps more willingly utilized, alongside their associated standards. That is, both the archives and their associated standards may be more likely to achieve greater ‘buy-in’ from their respective research communities than would be the case with a more centralized structure (Poldrack et al., 2024). Also, a diversity of archives can reach a diversity of users, propagating standards among various fields and institutions with varying levels of resources.

Building on these advantages, the BRAIN Initiative’s distributed archive ecosystem also offers distinct future opportunities, as more data are generated, analytical tools are refined, and data-sharing and reuse grow more commonplace. One advantage is the possibility for decoupling neuroscience research grants from infrastructure building. “[I]n some ways, almost every R01 ends up having a little aim with the resource that they — incredible resource — but they’re hosting it and they bear the brunt of supporting that, which shouldn’t be the case. We want to reduce that burden to the R01 researchers…” one policymaker remarked (Interview 114). As the NIH’s 2023 DMS policy accelerates the rate of sharing, the BRAIN archives are likely to provide viable options for data that might otherwise have nowhere else to go, other than into small, individual investigator-hosted archives posing their own interoperability and stewardship challenges.

Additionally, the BRAIN Initiative archives offer raw material for network evolution, and thus for responding to the needs of a large and growing community. “Database development is like spinal cord development, right?” one policymaker noted (Interview 116).

“You overproduce neurons, here you’ve got a bunch of muscles and things that are ready to do work, but they’re sort of poorly coordinated. And at some point, they come out and they meet; the use cases start to meet, and most of the neurons die, right? Because most of them turned out not to be useful, or they were overengineered. But then, once the use cases come, you start to fine-tune the system for the things that people actually want to do. And need to do. As opposed to every possible thing that could be done. So, I think that’s a really good metaphor. …I think we're in an exciting time now where the connections have finally been made, right?...There’s going to be a lot of pruning, there’s going to be a lot of refinement. But that could not happen until people actually were motivated to try to use these things.” (Interview 116)

The optimal ‘solution architecture’, as another policymaker put it, can emerge as the BRAIN Initiative and brain science evolve, from the possibilities offered by the current network (Interview 114).

There also is ample room for building further paths of interoperability, whether between standards or archives. “[T]hat may not mean shutting down other databases…” one neuroscientist noted (Interview 308). Indeed, rather than stabilizing at a middle ground with fewer archives, in the future the BRAIN Initiative data-sharing infrastructure might expand (https://braininitiative.nih.gov/funding-opportunies/notice-special-interest-nosi-brain-initiative-developing-data-archive). Thus: “[Improving interoperability] may just be creating conduits between [the archives], so it’s a one-stop shopping where you get data from all of them concurrently” (Interview 308). As the leaders of NWB emphasized in 2022: “Creating a coherent model of how the brain works will require synthesizing data generated by these heterogeneous experiments” (Rübel et al., 2022), whether within or across archives.

## Discussion

The BRAIN Initiative’s data-sharing ecosystem illustrates a range of possible responses to recurrent issues in database design and organization (Jwa and Poldrack, 2022; Lin et al., 2020; Institute of Medicine (US), 2013; Subash et al., 2023). The distributed BRAIN ecosystem might on the surface seem to be a natural reflection of the diversity of neuroscience data. Yet, as noted above, the NIMH follows a centralized approach (https://nda.nih.gov/) (Lee and Majumder, 2022). The HBP also has a centralized repository, EBRAINS (https://ebrains.eu/) (Amunts et al., 2019). Moreover, there has been a tendency to centralize data in other fields, such as in genomics (Cook-Deegan et al., 2017; Cook-Deegan and McGuire, 2017). At least since the conclusion of the Human Genome Project, when DNA sequences were funneled into separate regional archives (Maxson Jones et al., 2018; Stevens, 2018), most complex human genomic data in the US has been channeled into dbGaP or (more recently for large datasets) the Genomic Analysis, Visualization, and Informatics Lab-space (AnVIL) (https://www.genome.gov/Funded-Programs-Projects/Computational-Genomics-and-Data-Science-Program/Genomic-Analysis-Visualization-Informatics-Lab-space-AnVIL). Thus, the degree of database centralization for any project is a policy choice, not an inevitability, and one that must rely on a balance of competing interests, goals, and continual reassessment of challenges, benefits, and opportunities.

Accordingly, the neuroscience data-sharing ecosystem should be viewed as malleable, both inside and outside the BRAIN Initiative. The meetings that led to BossDB were part of broader, ongoing conversations about how data sharing could be promoted and realized in the digital age (Choudhury et al., 2014; Insel et al., 2003; Poldrack and Poline, 2015; Poline et al., 2012; Sejnowski et al., 2014; Van Horn and Gazzaniga, 2002; Van Horn and Toga, 2009; Teeters et al., 2008). Data sharing also has long been a priority among a subset of the neuroimaging (MRI) community, including within initiatives focused on disease research (Borghi and Van Gulick, 2018; Mennes et al., 2013; Poline et al., 2012). A few more specific examples include the Alzheimer’s Disease Neuroimaging Initiative (ADNI) (Jones‐Davis and Buckholtz, 2015), SchizConnect for schizophrenia (Ambite et al., 2015), and the Image and Data Archive (IDA) within the LONI at USC, which “started managing neuroimaging data for multi-centered research studies in the late 1990’s” (https://ida.loni.usc.edu/login.jsp). For over two decades, these leaders within neuroimaging have promoted data sharing for similar reasons to the BRAIN Initiative, emphasizing the goals of improving data quality and promoting reproducibility.

Additionally, existing literature has examined some of the challenges, alongside the benefits, of data sharing in neuroscience generally (i.e., Milham et al., 2018; Poline et al., 2012; Wiener et al., 2016; Rahimzadeh et al., 2023). This work has emphasized that major barriers to sharing include establishing and maintaining paths of data and archive interoperability and stewardship, such as sustainability and informed consent. Several data-sharing projects and supporting institutions, some founded before the start of the BRAIN Initiative, also interact closely with the BRAIN Initiative. These include the Neurosciences Information Framework (NIF) (https://neuinfo.org/) and the INCF, founded in 2006 and 2005, respectively (Poline et al., 2022; Poline et al., 2012; Sandström et al., 2022). Following meetings involving academia, industry, government, and the non-profit sector, the IBI was formed in 2018 (https://www.internationalbraininitiative.org/about). Such collective efforts are making neuroscience data FAIRer, for instance by streamlining collaborations and promoting adherence to data-sharing guidelines.

In turn, and in time, the relative benefits and drawbacks of centralized versus federated models in neuroscience data sharing also will become clearer. For example, there is the question of the value of any one archive. How can archives demonstrate and enhance their contributions, helping funders to determine how best to use their resources?

Perhaps some archives should indeed cease to exist, if they are not utilized or are inefficient (OECD Global Science Forum, 2017). The NIH Strategic Plan for Data Science states the intention to support the development of “standard use, utility, and efficiency metrics [for archives] and review expectations for data resources and tools” (https://datascience.nih.gov/nih-strategic-plan-data-science). Relatedly, a 2021 analysis employing interviews with representatives of 13 NIH repositories identified three categories of metrics for evaluation of an archive’s value: user behavior, scientific contribution/impact, and repository operations (NIH Metrics & LifeCycle Working Group and Metrics for Repositories (MetRe) Working Group, 2021). The NIH’s 2020 guidance, for data producers looking to select a repository, also lists among desirable characteristics for an archive “the ability to measure attribution, citation, and reuse of data (i.e., through assignment of adequate metadata and unique PIDs)” (https://grants.nih.gov/grants/guide/notice-files/NOT-OD-21-016.html). This feature also could benefit data contributors, helping to establish the value of an individual’s data-sharing efforts within the field.

The leaders of a few archives and datasets, such as at OpenNeuro, have managed to track information about reuse and resulting publications over time, and have estimated the savings realized through data sharing in terms of numbers of clinic hours and research costs (Markiewicz et al., 2021; Milham et al., 2018). Markiewicz et al. found that from 2018 to 2021, data reused from OpenNeuro resulted in 165 publications, with the volume increasing sharply over that period. Areas of contribution ranged from basic neuroscience (i.e., anatomical changes in the brain distinctive to pain syndromes) to the development and refinement of software tools (i.e., improvement of fMRIPrep preprocessing workflow). Collectively, as of 2021, these publications had generated over 1329 citations. In terms of research savings—that is, the cost were the reused data to have been generated de novo—Markiewicz et al. estimated nearly $21 million, using conservative assumptions (Markiewicz et al., 2021).

Recent announcements also have explicitly signaled the NIH’s interest in funding evaluation metrics. Examples include: “Notice of Special Interest (NOSI): Support for existing data repositories to align with FAIR and TRUST principles and evaluate usage, utility, and impact” (NOT-OD-23-044); “Enhancement and Management of Established Biomedical Data Repositories and Knowledgebases (PAR-23-237)”; and “Early-stage Biomedical Data Repositories and Knowledgebases (PAR-23-236)”.

However, such data remain difficult to obtain and are few and far between. As uses and citation practices vary, rigorous bibliometric analyses have necessitated full-text searches in Google Scholar followed by manual review (as with the OpenNeuro example above). Publication counts also tend to be lagging indicators of the scientific utility of a dataset. Furthermore, consensus standards for measuring the impact of a given dataset, such as counting the publications resulting from reuse, do not yet exist, even as various groups, including within the NIH, are studying the problem. Proposals for new author-level metrics of scientific achievement that integrate data sharing (i.e., the ‘S-index’ or ‘SCIENCE-index Augmented’) also have not yet been widely adopted (Emanuele and Minoretti, 2023), and the development and adoption of standard archive-level metrics to evaluate usage, utility, and impact remain a work in progress.

In sum, at present, the value of any one archive within a distributed ecosystem is challenging to measure quantitatively. Furthermore, without adequate redundancy, the disappearance of any single archive could cause instabilities in the entire system. Some redundancy is built into the BRAIN Initiative data-sharing ecosystem, for instance in the ability to store electrophysiology data in DABI, DANDI, or OpenNeuro and the extensions of the BIDS standards to EEG, iEEG, and MEG (Holdgraf et al., 2019; Niso et al., 2018; Pernet et al., 2019) in 2018 and 2019. But the ecosystem will continue to evolve, and it will require a sustainability plan. As scientists, policymakers, data stewards, and other interested parties consider the future of this data-sharing infrastructure, some of the primary considerations will need to include balancing needs and priorities in the areas of flexibility, sensitivity to innovation, diversity of reach, and coordinated management and sustainability.

## Materials and methods

Data for this manuscript were generated using mixed methods, including interviews with stakeholders and a landscape analysis of relevant policies, guidance documents, and data repositories.

To understand how and when researchers are required to share data, relevant policies and guidelines developed by the NIH were reviewed, including the BRAIN Initiative’s Data Sharing Policy (NOT-MH-19-010) and the new NIH-wide DMS policy (NOT-OD-21-013).

We also explored how each of the seven BRAIN Initiative data archives is structured and how it functions. One team member (SI) created accounts for each of the archives to ascertain data upload procedures (including formatting), what data were available for download, and available tiers of access to datasets (i.e., open or controlled access). Additional information was generated by a review of the literature including technical articles describing the archives (i.e., Delorme et al., 2022; Duncan et al., 2023; Hider et al., 2022; Markiewicz et al., 2021; Rübel et al., 2022; Subash et al., 2023).

Once user accounts were established at each archive, SI used an Excel spreadsheet to systematically capture defining characteristics of each archive, including similarities and areas of variation among them, as well as connections between them. Variables of interest included: privacy (defined as pathways of access to data, including whether accounts were required to upload, download, view, and/or analyze, and open/public and/or controlled access options); approaches for data backup and maintenance; data upload and download processes; accepted data types and files; analysis tools; and any external repository or resource interactions (i.e., links to access archive datasets from outside resources). In some cases, outreach was made to archive contacts through the websites’ Help or Contact Us functions. Communications with these members of the archives’ staff helped confirm understandings of how the archives worked, usually clarifying or corroborating information available from archive websites and publications.

Finally, we include data from semi-structured interviews (*n* = 34) with individuals who were recruited based on their work and expertise as BRAIN and non-BRAIN scientists (*n* = 9), data stewards (*n* = 4), law and ethics scholars and policymakers working in government and not-for-profit institutions (*n* = 10), patient advocates (*n* = 5), and industry representatives (*n* = 6). Interviews were conducted virtually as part of the BRAINshare project to explore perspectives on data sharing from human neuroscience studies, including barriers and facilitators of data sharing. Interviews were conducted in English and audio-recorded. Participants were offered a $50 electronic gift card upon completion of the interview. De-identified transcripts were analyzed using thematic analysis (Creswell and Poth, 2018). A codebook was developed deductively from the interview guide, which was revised with codes generated inductively. All transcripts were coded by a primary coder (KMJ, MAM, or AD), and a secondary coder (JOR, MAM, or AD) reviewed each transcript. Any discrepancies were discussed and resolved by consensus. For this manuscript, we reviewed data that were coded as related to credits, rewards, and incentives for sharing human neuroscience data, as well as technical aspects of sharing. Selected quotations represent salient issues identified. Dedoose, a web application for managing qualitative research, was used for the coding process (version 9.0.82, 2022, Los Angeles, CA: SocioCultural Research Consultants, LLC). This study was approved by the Baylor College of Medicine Institutional Review Board (protocol H-49969).
